# Therapeutic potential of interferon-gamma in tuberculosis

**DOI:** 10.5599/admet.1078

**Published:** 2022-02-14

**Authors:** Svetlana A. Berns, Julia A. Isakova, Polina I. Pekhtereva

**Affiliations:** 1 Federal State Institution National Medical Research Center for therapy and Preventive Medicine of the Ministry of Healthcare of the Russian Federation, svberns@yandex.ru; 2 SPP “Pharmaclon” Ltd., Moscow, Russia, julia.is.alex@gmail.com; 3 SPP “Pharmaclon” Ltd., Moscow, Russia, pekhterevapolina@gmail.com

**Keywords:** Interferon gamma, MRD-TB, immunotherapy, IFN-γ, complex treatment

## Abstract

Tuberculosis is one of the critical health problems worldwide. The search for ways to improve the results of tuberculosis treatment and overcome drug resistance lies in understanding the pathogenesis of the development of the infectious process. The interferon system, particularly the role of interferon-gamma, has been identified as the main link in the immune response in tuberculosis. The clinical efficacy of interferon-gamma has been studied and evaluated in clinical trials since the end of the last century. There was obtained evidence of the clinical efficacy of interferon-gamma as part of complex therapy. Recent experimental data make it possible to consider interferon-gamma as a promising therapeutic option for the treatment of multidrug-resistant tuberculosis as part of complex therapy worthy of further studies.

## Introduction

The problem of resistance of microorganisms to antimicrobial drugs and, consequently, issues related to the ineffectiveness of treatment remain one of the most urgent in medicine. The World Health Organization (WHO) developed and published in 2001 the WHO Global Strategy for Containing Antimicrobial Resistance, which recommends considering this problem as one of the priorities of national health systems [1]. The significance of the problem of antibiotic resistance is determined by the widespread prevalence of resistant microorganisms in inpatient departments and the wide access to drugs used for the treatment of multi-resistant strains [2]. A decrease in the effectiveness of therapy ultimately requires the use of more expensive drugs and an increase in the duration of treatment, which leads to hospitalization time increase, economic losses, and mortality risk [3].

Tuberculosis (TB) is a socially significant disease and the deadliest infection, continuing to be the most pressing challenge for the healthcare system around the world. According to WHO, TB is the ninth leading cause of death in the world, having been the leading cause of death from any single infectious agent, ahead of Human Immunodeficiency Virus (HIV) / Acquired Immunodeficiency Syndrome (AIDS), till 2020, when COVID-19 occupied this place [4]. In 2019, WHO estimates that 1.21 million HIV-negative people died from TB (less than 1.7 million deaths in 2000), and 208,000 deaths were reported among HIV-positive people [5]. Thus, global TB deaths have been reduced by 35 % from 2015 to 2020. The incidence of TB has decreased by 20 % over the same period. In this way, there are notable successes in the fight against this infectious disease, but so far, not enough to achieve the global goals set by the WHO [5].

Most TB deaths can be prevented with early diagnosis and appropriate treatment. Millions of people are diagnosed and successfully treated with TB each year, but there are still significant gaps in the detection and therapy of the disease. The situation may be aggravated by the epidemic threat of COVID-19, in particular, due to the redistribution of human, financial, and other resources not in favour of diagnosis and treatment of tuberculosis. The number of medical institutions providing inpatient and outpatient care for TB patients has decreased, and there are overlaps in data collection and reporting [5].

In conditions of unstable drug supply, underdeveloped infrastructure, and limited resources [6], the threat of drug-resistant TB remains. According to WHO estimates, in 2019, in the world, 3.3 % (95 % confidence interval (CI): 2.3–4.3 %) of newly diagnosed TB cases and 18 % (95 % CI: 9.7–27 %) of previously treated patients have been identified as resistant to rifampicin (RR-TB), the most effective first-line drug, or multidrug-resistant TB (MDR-TB). Among them, 20.1 % (95 % CI: 15.5-25.0 %) were also resistant to fluoroquinolones, the drugs of choice in establishing resistance to rifampicin or isoniazid. It is alarming that about half of these cases are registered in India, China and the Russian Federation [5].

The problem of overcoming drug resistance to TB is also complicated because chemotherapy itself has an antimicrobial effect and suppresses the immune system's activity and can aggravate the patient's immunological reactivity. Impaired immune response in TB patients can be corrected by immunotherapy. The correction of the immune status in MDR-TB patients is of particular importance because these patients initially have a worse prognosis and a more severe course of the disease [7].

In such conditions, the possibility of adjuvant immunotherapy, which can change the course of the disease and alleviate the patient's condition, gains a special role. One of the ways of such therapy is to regulate the body's immune response.

### Features of the formation of the immune response in bacterial infection

Returning to the pathophysiological background of the immune response in bacterial infection, it is known that when the pathogen enters the human body for the first time, the innate and adaptive immune response mechanisms get activated [8]. In case of repeated exposure to the same pathogen, i. e. upon reinfection, the removal of the pathogen can occur at a higher rate due to protective immunity and immunological memory [9].

The important role of interferons (IFNs) in the implementation of the immune response process was proven back long ago [10,11]. The search for new ways to overcome drug resistance in patients with MDR-TB aimed at the role of IFN as an immunomodulator in the complex therapy of TB. The IFN system includes the IFN cytokines themselves, transcriptional activators and repressors, cellular receptors and enzyme systems activated by them. IFNs increase the resistance of cells to infectious agents and propagate signals to other cells that have not previously had contact with either the antigen or IFN. All IFNs are divided into 3 types: IFN of type I (-α, -β, -κ, -ε, -ω, -τ) [12], type II (-γ) [13] and type III (-λ1-4, -£1-3) [14].

At an early stage of infection (several hours), IFNs of types I and II are produced as the first line of immune defense in order to attract the largest number of dendritic cells and macrophages to the site of injury, which trigger active phagocytosis and inactivate the pathogen. However, if there is a defect in the immune mechanism, attracted macrophages can promote infection, providing the microorganism with a target for intracellular growth and spread [15,16]. In this case, type I IFNs can contribute to the development of TB by inducing IL-10 and deactivating macrophages [17,18]. The immune defense of the first level will be broken and the cellular response to the antigen blocked. As a result, a latent form of TB may develop [19].

The optimal immune response is formed in a few days after infection. CD4+ and CD8+ effector T cells are directed to the affected area and start inducing type II IFN, strongly shifting the balance towards this class of cytokines [15] and reducing the risk of developing an active TB process [20]. It was found that CD4+ lymphocyte count below 100 cells/mm3 before treatment significantly increases the likelihood of an unfavourable outcome of generalized tuberculosis in HIV patients [21].

**Figure 1.**
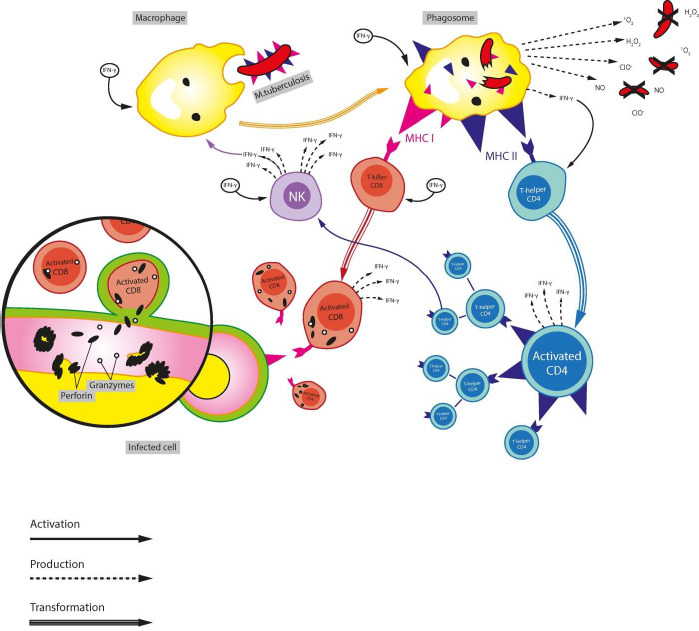
Mechanism of IFN-γ action via the immune cells

At least 10 genes are known to be associated with increased susceptibility to mycobacterial infection [22]. First of all, these are genes encoding IFN-γ and its receptors, as well as cellular factors STAT1 [23] and NFκB [24], directly related to the mechanism of IFN-γ action on the cell: antigen recognition, the realization of an innate immune response, and oxidative properties of macrophages [25], and necessary for protection against TB [26] and other infectious diseases, since they determine nonspecific pathways of action on the pathogen (Figure 1).

Disseminated forms of infections in children often arise due to congenital defects in the structure of the IFN-γ protein [27]. The deficiency of genes encoding protein chains of receptors is one of the most common mutations that cause increased susceptibility to mycobacterial infection. The IFNγR1 gene accounts for 39 % of mutations [28]. For reasons still unclear, IFNγR2 deficiency is much less common [29]. In patients with complete deficiencies of any of these genes, there is an absence or incomplete formation of granulomas and diffusely loaded macrophages [30].

### Features of the formation of bacterial resistance

The main mechanisms of the resistance development include genetic changes that ensure the "removal of the effect" of the antibiotic (change in the target, metabolism, method of removing the antibiotic from the cell), as well as the isolation of the microorganism from the drug (intracellular localization, biofilm formation) [31]. In the case of *M. tuberculosis*, the microorganism forms resistance due to the hydrophobic cell wall, β-lactamases, and mutations in the bacterial genes [7].

The immune system ensures the elimination of the pathogen primarily due to the strength of the cellular immunity by activating the phagocytosis.

It was shown that IFN-γ could affect each of the main aspects of the phagocytes activation, thereby playing an important role in the immune response [32-34]:

- activates macrophages, attracts them to the site of infection;
- induces the release of nitric oxide;
- serves as the only inducer in the cell of the synthesis of proteins of the second class histocompatibility complex (MHC class II) - an antigen-presenting complex for extracellular pathogens.

The primary feature of the functioning of phagocytes, which ensures the relationship of nonspecific and specific immunity, is the performance of the role of antigen-presenting cells. Antigen-presenting cells are located on the main routes of entry of antigens into the body (in the skin and the mucous membranes), from where, after capturing antigens, they migrate to the peripheral organs of the immune system, where they present antigens to lymphocytes. Phagocytes are the cells that directly kill bacteria through phagocytosis ("eating" them) and play one of the leading roles in the body's first immune response to bacterial infection. Hereditary and acquired disorders of phagocytosis can lead to severe pathologies of the functioning of the immune system [35].

Mechanisms of action of phagocytes:

- a phagocyte cell carries receptors on its surface that recognize an infectious agent (for example, the cell wall of a bacterium). When a phagocyte comes into contact with a bacterium, receptors on its surface bind to it, leading to the bacterium's absorption by the phagocyte.
- Release of reactive oxygen species and nitric oxide by phagocytes cause oxidative stress that destroys bacteria;
- Stimulation of other cells of the immune system through "antigen presentation" is a process in which phagocytes place fragments of pathogen proteins on the surface of their cells, thereby presenting these fragments to other cells of the immune system, developing an antigen-specific immune response [8,35-37].

It is important to note that at an early stage of infection, IFNs of types I and II are produced in equal concentrations, and IFNs of type I are able to reduce the expression of the major histocompatibility complex (MHC or MHC) of class II on antigen-presenting cells, induced by IFN of type II, preventing it action [38].

Thus, during the development of a bacterial infection, the effects of IFN-γ provide a relationship between the two most important links of the immune response of a macroorganism, enhancing the antigen-dependent immune response and stimulating the work of phagocytes.

An additional factor affecting the course of a bacterial infection is the ability of bacteria to inhibit the synthesis of pro-inflammatory cytokines. It has been shown that some microorganisms can specifically repress the synthesis of endogenous IFN-γ [39]. In a study by Fortes et al., it was demonstrated that patients infected with an antibiotic-resistant strain of Mycobacterium tuberculosis have a reduced level of endogenous IFN-γ compared to normal patients [40]. It has also been shown that the use of beta-lactam antibiotics can decrease the level of endogenous IFN-γ [41].

Thus, the IFN system and directly IFN-γ are key regulators of the body's immune response to the infection caused by TB. Therefore, additional exposure to exogenous IFN-γ can play the role of an inducer of the immune system under conditions of a reduced response to antibiotics and correct the endogenous cytokine deficiency.

Despite all the attractiveness of the idea, unfortunately, interest in the development has not yet been represented by widely deployed large-scale clinical trials (except for diagnostics use research). This is probably due to the limited number of medical products based on interferon-gamma presented on the world market. Nevertheless, in our view, the diagnostic and clinical value of the molecule will undoubtedly gain momentum in the nearest future.

### Clinical efficacy of interferon-gamma in the treatment of tuberculosis

On the open resource https://clinicaltrials.gov/ 99 clinical trials with keywords “interferon-gamma” and “tuberculosis” were found, 15 of which are in active status, 64 are completed, 20 are cancelled or have an unknown status. Out of 64 completed studies, 16 studies have published results. Current research is mainly concerned with the diagnostic use of the interferon-gamma secretion test to assess the immune status of the body [42].

The therapeutic activity of IFN-γ against TB and MDR-TB has been intensively studied since the end of the last century; a number of clinical studies have demonstrated the effectiveness of IFN-γ against TB, including MDR-TB [43-49].

A meta-analysis that pooled data from 9 clinical trials showed a statistically significant effect for IFN-γ as an adjunct drug for TB [43]. The studies of nebulized IFN-γ showed statistically significant benefits as a result of negative sputum conversion and chest radiographs: the pooled relative risk (RR) for conversion was 1.97 (95 % confidence interval (CI) 1.20-3.24, p = 0.008) after 1 month of treatment, 1.74 (95 % CI 1.30-2.34, p = 0.0002) after 2 months of treatment, 1.53 (95 % CI 1.16-2.01, p = 0.003) after 3 months of treatment, 1.57 (95 % CI 1.20-2.06, p = 0.001) after 6 months of treatment, and 1.55 (95 % CI 1.17-2.05, p = 0.002) at the end of treatment; the pooled RR for chest radiographs was 1.38 (95 % CI 1.10–1.17, p = 0.006) at the end of treatment [44]. A randomized controlled trial with aerosol and subcutaneous injected IFN-γ showed a significant reduction in fever symptoms, wheezing and night sweats compared with the control group after one month of treatment [46]. For intramuscularly administered IFN-γ, the meta-analysis included three studies that showed significant improvement in negative sputum conversion after two months of treatment [43]. Intramuscular injection of IFN-γ also showed a beneficial effect in treating pulmonary MDR-TB [45]. The authors concluded that adjuvant therapy with IFN-γ, especially in an aerosol form, may be beneficial for TB patients.

The results of another randomized clinical trial published in 2016 also confirmed the high clinical efficacy of IFN-γ as part of the complex therapy for TB. This study evaluated the clinical efficacy of IFN-γ in the intensive phase of complex treatment of newly diagnosed patients with destructive pulmonary TB with bacterial excretion. The dynamics of laboratory and immunological parameters were also assessed. The study included 60 patients (the study group included 30 newly diagnosed patients who received 500,000 IU of IFN-γ intramuscularly every other day, for a month in addition to standard therapy; control group - 30 patients who received only standard therapy). The predominant clinical form was infiltrative, diagnosed in 80 % of patients in both groups (p> 0.05). Clinical efficacy was assessed in patients who received IFN-γ after two months of the intensive phase of treatment. According to the results of a control X-ray examination, cavities in the lungs closed in 23 % of cases, and by four months, this indicator was 50 %. While in patients in the control group, the closure of cavities in the lungs was 10 %, in the study group, this indicator was three times higher (37 %) (p <0.05). Abacillation occurred in 90 % of patients receiving IFN-γ. A month later, the normalization of indicators of the clinical analysis of blood occurred in 70 % of patients. In the group of patients who additionally received IFN-γ, the number of CD4+ lymphocytes increased to 41 %, exceeding the initial level by 1.7 times (p <0.001). The authors of the study concluded that the addition of immunomodulatory therapy is a significant addition to anti-TB drugs in terms of pathogenesis. The use of IFN-γ in the complex treatment of patients with destructive pulmonary TB with bacterial excretion has shown its high efficiency in the intensive phase of chemotherapy, accompanied by an increase in patients' quality of life. The results of the study indicate a significant improvement in the quality of life based on the assessment according to the methodology recommended by the WHO. After complex treatment, when studying the quality of life of patients in the main group, there was a positive trend in aspects characterizing social relationships and the psychological sphere, as well as reflecting the physical and mental health of the patient. It should be noted that the addition of IFN-γ to anti-tuberculosis therapy was not accompanied by a significant increase in the number of adverse reactions (20 % vs. 17 % in the control group) (p> 0.05). All adverse reactions were removable and stopped by pathogenetic methods of concomitant treatment [48].

One more clinical example was published in the scientific literature about the experience of IFN-γ medical use in MDR-TB. The treatment of MDR-TB of the brain in a patient with acute lymphocytic leukemia, after five months of adjuvant therapy using subcutaneously injected IFN-γ, showed a positive trend with the achievement of complete remission after 12 months of therapy [47].

In another study evaluating the effects of IFN-γ in MDR-TB, it was found for resistant forms of TB that there is an increase in the basal and IL12/IL27-induced secretion of IFN-γ by blood lymphocytes in vitro, with the maximum severity of changes in the disseminated form of the disease. Hypersecretion of IFN-γ in vitro is associated with a low content of CD3-IFN-γ+ lymphocytes and an increase in the proportion of CD3-IFN-γ+ against the background of general T-lymphocytopenia [50].

A recent study to assess the effects of IFN-γ on macrophage activity in overcoming MDR-TB drug resistance demonstrated the high efficacy of IFN-γ as an adjuvant therapy for MDR-TB. The authors of the study draw encouraging conclusions about the prospect of using IFN-γ as a part of adjuvant therapy for MDR-TB. It increases the activity of macrophages against Mycobacterium tuberculosis and improves the activity of macrophages treatment outcomes in patients with poor response to therapy [7].

The use of exogenous IFN-γ in adjuvant therapy, especially in the case of diseases caused by microorganisms resistant to antibiotics, can have a significant effect on the course of the disease and accelerate the patient's recovery, contributing to earlier abacillation of sputum.

### Assessment of the economic feasibility of using interferon-gamma in the treatment of tuberculosis (experience of the Russian Federation)

As a socially significant disease, TB poses a significant social and economic burden on the healthcare system around the world [51]. The economic burden of TB is conditioned by several components: direct economic losses associated with medical costs and indirect economic losses of the country's gross domestic product due to the loss of “human capital”. The costs of drug therapy account for more than half of the direct medical costs of TB treatment [52]. In this regard, when adding new drugs to TB treatment regimens, not only clinical indicators of the effectiveness of therapy should be taken into account, but also their impact on the health economics. Assessment of health technologies when deciding on the inclusion of medicines in the drug supply system is a mandatory component of health systems in all countries of the world, regardless of economic development, morbidity and mortality. In the Russian study published in 2018 [53] on the assessment of the cost-effectiveness for IFN-γ, a positive impact on the budget from the use of IFN-γ as a part of complex TB therapy was demonstrated due to reducing the duration of hospitalization. As a result of the behavioural pharmacoeconomic analysis, it was shown that IFN-γ contributed to an increase of the duration of quality life by 2.1 QALY, and its use will be accompanied by savings in the budget of the Russian Federation in the amount of up to 27 % of funds for the treatment of this group of patients [53].

## Results and Discussion

Analysis of data from preclinical and clinical studies shows that the inflammatory cytokine IFN-γ occupies an important place in the pathogenesis of bacterial infections, including TB.

The significance and potential of the use of interferons are discussed in all areas of phthisiology: diagnosis, therapy, and prevention of TB [54]. For instance, the main alternative to tuberculin skin test widely used for diagnostic purposes is IFN-γ release assays (IGRA) test. It measures the ex vivo production of IFN-γ from T-cells stimulated with the TB-specific antigens. The test doesn’t have cross-reactivity to BCG and nontuberculous mycobacteria showing higher specificity keeping the same level of sensitivity [55-57].

Preclinical data confirm the enhancement of the cellular component of immunity when using IFN-γ in combination with the recombinant BCG vaccine (Bacillus Calmette - Guérin (BCG)) [58]. The use of IFN-γ as a means of preventing infection, as well as the transition of the latent phase of TB to the active form, requires the study of this agent in clinical trials of vaccines and other therapeutic drugs [20].

The greatest amount of evidence and data today is obtained in the direction of the use of IFN-γ as a therapeutic option in the complex therapy of pulmonary TB. It has been shown that IFN-γ plays a significant role in the pathogenesis of TB [15,59], is a marker of type 1 Th-helper lymphocytes [55], and is considered a key immunomodulatory agent for the maintenance adjuvant therapy of TB patients.

Many factors play a role in the infectious process and outcomes of TB, but the most important of them is the activity of the body's immune system and its ability to resist infection. The human immune system is able to control it. Weakness of the immune response is the main condition for the development of the disease and subsequently for the prolonged course of the infectious process against the background of antibiotic therapy. In patients with immunodeficiency, the treatment of the disease is always difficult, even with maximum doses of drugs as part of complex antibiotic therapy [6]. In conditions of bacterial resistance to antibiotics, the role of the immune response in the fight against infection and the stress on the immune system increases dramatically. S.H. Kaufman et al., in their review published in 2014 in the Lancet [60], focused on host-directed therapies defining such strategy “especially beneficial for patients with multidrug-resistant tuberculosis”. The use of traditional anti-tuberculosis drugs only is no more a sufficient tactic to achieve the target [60].

## Conclusions

Understanding the pathogenesis of the development of the infectious process and the role of IFN-γ in the activation of the immune system in TB, made it possible to identify IFN-γ as a promising therapeutic option for improving treatment results and overcoming the development of bacterial resistance. The clinical efficacy of IFN-γ against TB has been studied in clinical trials for over 20 years. The clinical experience and medical application of IFN-γ accumulated in the course of long research allow us to recommend it as a safe and effective therapeutic agent in a complex, comprehensive TB treatment, not only from the standpoint of evidence-based medicine but also from the point of view of health economics. For the full disclosure of the therapeutic potential of IFN-γ in overcoming multidrug resistance of TB, further study of its effectiveness in controlled clinical trials is necessary.
